# Optimization of Antimicrobial Use for Sepsis in Calves: Bayesian Evaluation of Existing and Novel Sepsis Scores

**DOI:** 10.3390/ani15040586

**Published:** 2025-02-18

**Authors:** Mathilde Laetitia Pas, Jade Bokma, Filip Boyen, Laurens Chantillon, Donatienne Castelain, Justine Clinquart, Stan Jourquin, Bart Pardon

**Affiliations:** 1 Department of Internal Medicine, Reproduction and Population Medicine, Faculty of Veterinary Medicine, Ghent University, Salisburylaan 133, 9820 Merelbeke, Belgium; jade.bokma@venhei.be (J.B.); laurens.chantillon@ugent.be (L.C.); donatienne.castelain@ugent.be (D.C.); justine.clinquart@ugent.be (J.C.); stan.jourquin@ugent.be (S.J.); bart.pardon@ugent.be (B.P.); 2 Veterinary Practice Venhei, Geelsebaan 95-97, 2460 Kasterlee, Belgium; 3 Department of Pathobiology, Pharmacology and Zoological Medicine, Faculty of Veterinary Medicine, Ghent University, Salisburylaan 133, 9820 Merelbeke, Belgium; filip.boyen@ugent.be

**Keywords:** antimicrobial stewardship, bacteremia, Bayesian latent class model, blood culture, systemic inflammatory response syndrome

## Abstract

Early recognition and rapid appropriate antimicrobial treatment is essential for survival of sepsis. To date, it is unclear which sepsis score should be used for an early diagnosis in calves. The objective of this study was to evaluate two existing scores (Trefz and Fecteau), three novel calf sepsis screening models (CSS, CSS_A_, CSS_B_), and blood culture as diagnostic test for sepsis, using Bayesian latent class evaluation. A total of 131 sick calves were included in this study. Sepsis prevalence was 45%, 27%, 56%, 47%, and 55%, when using the Trefz score, Fecteau score, CSS, CSS_A_, and CSS_B,_ respectively, and 22% had a relevant positive blood culture. The newly established models CSS (including ≥2 SIRS-criteria and abnormal mental state) and CSS_B_ (alternative model CSS for practice, substituting abnormal leukocyte count with mucosae) had the highest sensitivity, with 86% and 84%, respectively, and could be interesting screening tests for sepsis. Sensitivity of the existing Trefz and Fecteau score was 70% and 35–39%, respectively. The presented new sepsis scoring systems have potential as screening tests to identify calves at risk, after which a calf-side diagnostic test is advised to confirm the diagnosis. Its use might aid in the rationalization of antimicrobial use in critically ill calves.

## Simple Summary

Sepsis is the presence of a pathogen in the bloodstream, causing the body to react to fight the infection. However, sepsis is a difficult condition to recognize. This study looked at ways to identify sepsis in calves, comparing both previous existing and newly suggested sepsis. The most sensitive sepsis score appeared to be a newly proposed model, which involved looking for abnormalities in heart rate, breathing frequency, temperature, white blood cells, and abnormal mental state. An alternative version of this sepsis score, in which white blood cells are replaced by abnormal mucosal membrane color in SIRS, could serve as a recognition method for veterinarians in practice, because the sensitivity of that test is only slightly lower. The new sepsis scores could help identify calves at risk of sepsis. After this screening, a quick diagnostic test at the farm is recommended to confirm the sepsis. The suggested screening systems might help ensure antibiotics are used more wisely in sick calves.

## 1. Introduction

Sepsis, defined as a life-threatening organ dysfunction caused by a dysregulated host response to infection, affects welfare and leads to production losses and the use of critically important antimicrobials (CIA) in calves [1,2,3,4,5,6,7]. To improve survival, an early diagnosis and broad-spectrum antimicrobial treatment is necessary, for which clinicians often resort to CIA [8,9]. However, early diagnosis is a huge challenge in both humans and calves, and the pressure to reduce the use of antimicrobials in animals, especially CIAs, complicates appropriate treatment. Bacteremia in critically ill calves, and thus assumed septic calves, has been shown to significantly increase mortality risk, emphasizing its importance [6]. However, blood culture, the reference standard for bacteremia, entails an extensive period before results are known and has a limited sensitivity, making it an insufficient diagnostic tool for this disease [5,10]. Extensive research is being conducted on specific laboratory markers (e.g., procalcitonin) to confirm the diagnosis and address the limitations of blood cultures [11,12,13,14,15,16], even including metabolomics-based approaches [17]. While this research is crucial, particularly for the rational use of CIA, currently, the absence of a proper consensus definition and a sensitive primary screening method for sepsis in calves remains a first major challenge. Multiple studies examined risk factors and clinical signs predicting bacteremia and septicemia [1,3,5,18]. Both Fecteau et al. [1] and Lofstedt et al. [3] constructed predictive scoring models to determine the sepsis risk. In addition, a single study adapted the in human medicine previously used systemic inflammatory response syndrome (SIRS) criteria to calves [19]. However, most of these proposed diagnostic methods for sepsis lacked proper external validation. Plus, in the latter definition, abnormal mental state, considered an important indication of sepsis in human medicine, and previously associated with bacteremia and septicemia in calves, is absent [5,7,18]. Thus, a combination of abnormal SIRS-criteria with abnormal behavior might be an interesting possibility for a screening alternative. However, as SIRS includes information on white blood cell count, other more basic alternatives, such as abnormal color of the mucosal membranes, might be interesting for practical use [2]. Therefore, the objective of this study was to evaluate the Fecteau and Trefz scores, as well as novel sepsis scoring models in sick calves, comparing their diagnostic accuracy with blood culture as an initial diagnostic screening for sepsis, using a Bayesian latent class model.

## 2. Materials and Methods

### 2.1. Study Design and Study Sample

A retrospective diagnostic test study was performed to evaluate sepsis scoring methods in comparison with blood culture for diagnosing sepsis in calves. The study included sick calves presented to the clinic for ruminants of Ghent University or examined on farms (*n* = 11 farms). Sick calves included both critically ill calves with signs of systemic disease (such as severe respiratory, cardiovascular, or neurological symptoms) and non-critically ill calves. The latter group comprised calves with evident morbidities, such as diarrhea, pneumonia, and omphalitis, but without signs of systemic illness, meaning < 2 SIRS criteria, a normal mental state, and no significant cardiovascular or respiratory abnormalities. In previous studies [5,6], we defined sepsis as the presence of critical illness combined with a relevant positive blood culture. While critically ill animals represent the primary at-risk population for sepsis, this definition likely underestimates true cases due to the imperfect sensitivity of blood cultures. In this study, we did not apply a strict definition of sepsis, as our primary goal was to optimize sepsis diagnosis using Bayesian analysis while minimizing the risk of overlooking important cases. Further inclusion criteria were the availability of blood culture results and an age ≤ 3 months at the moment of sampling. The study period spanned from 2019 to June 2024. Animals with incomplete data for any of the diagnostic tests were excluded, resulting in the inclusion of 131 calves in the final dataset. For sample size estimation, a minimum of 103 calves was required based on an estimated prevalence of 30%, a null hypothesis value of 0.70, an alternative hypothesis value of 0.90, and a power of 80%, as recommended by Bujang and Adnan [20]. To ensure meticulousness, the authors chose a larger sample size appropriate for a diagnostic test rather than a screening test. The sampling performed in this study was approved by the ethical committee of the Faculties of Veterinary Medicine and Biosciences of Ghent University under license number EC 2021-023. The study was conducted following the STARD-BLCM guidelines [21].

### 2.2. Clinical Examination

Information on demographics of the calves (breed, age, sex) as well as calves’ medical history regarding prior treatments were included in the database. Clinical information in the database included rectal temperature (°C), heart rate (beats per minute), respiratory frequence (breaths per minute), information on behavior (mental state and posture), color of the oral and conjunctival mucosal membranes, fecal score, umbilical thickness and painfulness, hydration status (skin pinch and enophthalmos), scleral vessels, presence of hypopyon, nasal discharge, presence of arthritis and soft tissue abscesses, and other striking abnormalities. Clinical examination was performed by multiple clinicians (*n* = 8), all trained by the same senior supervisor. Ultrasound was performed on the lungs, intestines, liver, spleen, and umbilical structures as previously described [5,6]. As enteritis, omphalitis, and pneumonia are the most well-known conditions leading to sepsis, information on these morbidities was recorded in the dataset [2,4].

Blood for hematology (whole blood count) was sampled out of the jugular vein with a 4 mL EDTA tube and analyzed using an IDEXX ProCyte Dx Hematology Analyzer^®^ (IDEXX Europe B.V., Hoofddorp, The Netherlands). Blood cultures were aseptically sampled, similar as previously described [22].

### 2.3. Bayesian Latent Class Model

In the absence of a traditional gold standard diagnostic test for sepsis in calves, a Bayesian latent class analysis is the ideal statistical approach to counter this practical limitation for estimating test accuracy and disease prevalence [23]. In this study, blood culture was compared to multiple sepsis scoring models to diagnose sepsis.

#### 2.3.1. Definitions of Diagnostic Tests Included in Bayesian Latent Class Model

**Blood culture** positive calves were defined as calves from which a likely relevant pathogen was isolated from the blood culture. Excluding likely contaminants was conducted as previously described [22]. In short, pathogens were considered a true pathogen when they were isolated from two blood culture flasks and were a member of the Enterobacterales or a well-known significant bovine pathogen. Bacteremic calves were, in this study, defined as calves from which an isolate was obtained that was either a member of the Enterobacterales or an important bovine pathogen, or the same pathogen was isolated from two separate blood cultures.

The first included sepsis scoring model, the **Trefz score**, was based on a definition by Trefz et al. [19]. In this definition, an animal with minimum of two positive SIRS-criteria, adapted from human medicine, was considered to have sepsis when presented in combination with hyperemia of mucous mucosae, mucosal or subscleral bleedings, or the presence of hypopyon. The SIRS criteria values adapted to calves included an abnormal leukocyte count (normal reference interval, 5–12 G/L), abnormal rectal temperature (normal reference; 38.5–39.5 °C), tachycardia (>120 bpm), and tachypnea (>36 bpm) [19]. 

The second sepsis score, the **Fecteau score**, included the presence of a focal infection (hypopyon, septic arthritis, soft tissue abscess, or mucopurulent nasal discharge), age, hydration status, scleral vessel injections, mental state, umbilical clinical state, and fecal consistency, which were scored according to the model proposed by Fecteau et al. [1]. Calves were scored as septicemic when above the 40.8% (sepsis score > 3.3) probability, the optimum cut-off to predict bacteremic calves according to Fecteau et al. [1].

**Figure 1:**
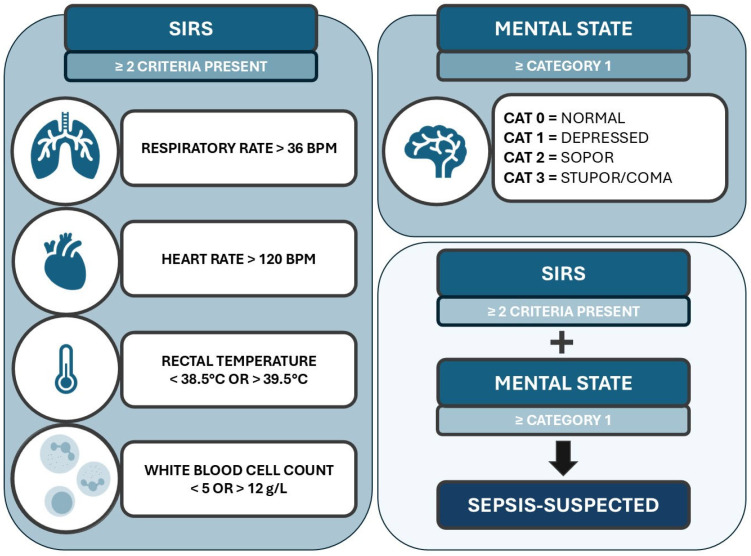
Visual representation of the calf screening score (CSS), in which the combination of ≥2 SIRS criteria and abnormal mental state were considered to identify sepsis-suspected calves. This model is proposed for its use in a hospital setting.

The third sepsis scoring model, named **calves sepsis screening** (**CSS**), is the first proposed new alternative, and is the combination of the previously mentioned SIRS-criteria (abnormal leukocyte count, abnormal rectal temperature, tachycardia, and tachypnea) and the presence of an abnormal mental state. The reference values used for SIRS were the same values suggested by Trefz et al. [19]. Mental state was scored similar as in Pas et al. (2024), which was adapted from the scoring by Fecteau et al. [1,5]. When behavioral scoring was ≥1, this was considered an abnormal mental state. When both an abnormal mental state and ≥2 SIRS criteria were present, the calves were considered septicemic. Considering CSS includes leukocyte count results, this model cannot be used calf-side on farms but could be used in a hospital setting. The CSS model was visualized in Figure 1.

**Figure 2:**
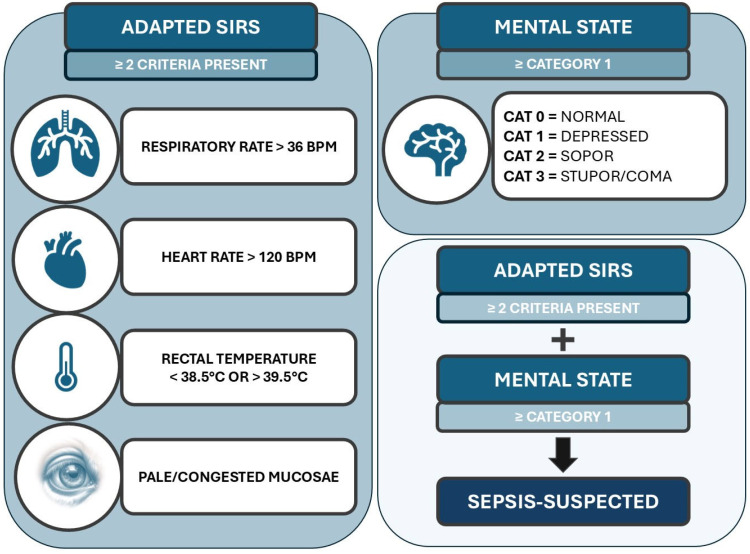
Visual representation of the adapted calf screening score (CSS_B_) for application in first-line practice. SIRS-criteria were adapted. Abnormal white blood cell count was substituted by abnormal mucosal color to function as a calf-side test. The combination of ≥2 adapted SIRS criteria and abnormal mental state were considered to identify sepsis-suspected calves.

For this reason, two additional adaptations on CSS were constructed as calf-side alternatives, neither requiring any blood tests. In **calf sepsis screening** A (**CSS_A_**), the leukocyte count was eliminated, thus only including three SIRS criteria: abnormal rectal temperature, tachycardia, and tachypnea. A visual representation of this screening model can be found in the supplementary information (Figure S1). At least 2/3 SIRS criteria and abnormal mental state had to be present for the calves to be sepsis-suspected. An alternative **calf sepsis screening B** (**CSS_B_**) was added, replacing the leukocyte count with abnormal mucosal color. Thus, in this model, when behavioral scoring was ≥1, and when ≥2 adapted SIRS criteria (abnormal rectal temperature, tachycardia, tachypnea, and abnormal mucosal color) were present, the calves were considered to have sepsis. A visual representation of this screening system can be found in Figure 2.

**Figure 3:**
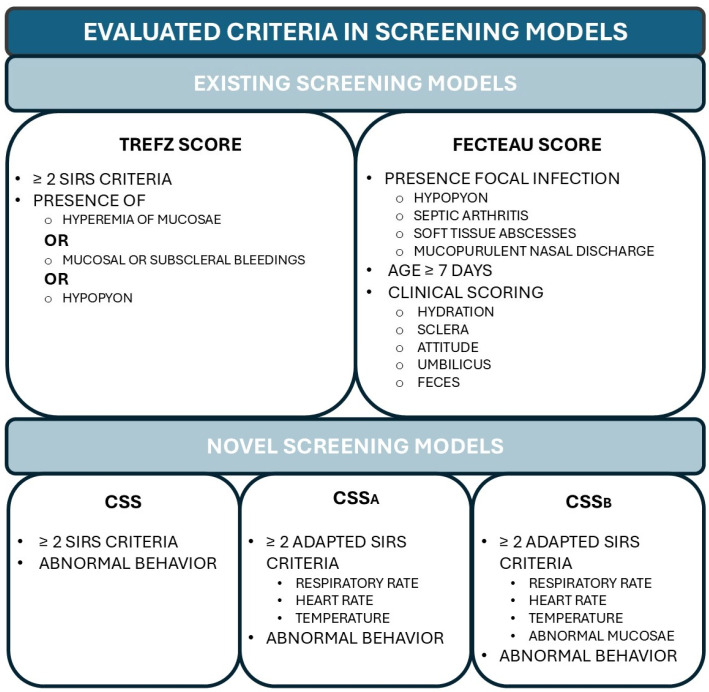
Condensed overview of the evaluated screening models for sepsis in calves and their respective criteria, including two previously described models (Fecteau et al. [1] and Trefz et al. [19]), as well as three new screening models (CSS, CSS_A_, CSS_B_).

A condensed overview of the evaluated five different screening models for sepsis and their evaluated criteria can be found in Figure 3.

#### 2.3.2. Statistical Analysis

A database including all calves meeting the inclusion criteria was established in a worksheet (Excel, Microsoft Inc., Washington, DC, USA). This database contained all relevant clinical information, plus information on blood culture results of the calves. Each calf was scored binary as septic (1) or non-septic (0) according to the abovementioned scoring systems and according to the described guidelines for relevant bacteremia (Section 2.3.1). The obtained binary data were transferred to WINBUGS for Bayesian latent class analysis (v1.4.3.; MRC Biostatistics Unit, Cambridge, UK) to determine the sensitivity and specificity of both clinical scoring models and bacteremia in this population of sick calves. The Bayesian analysis was chosen to address the absence of a reference standard test. This method avoids assuming the superiority of any single test by establishing its own probabilistic definition of the studied outcome [23]. The study population was considered one single population, as calves from multiple farms throughout Belgium were sampled, thus a one population 3-test Bayesian latent class analyses was conducted. As the sepsis scores were based on clinical signs (with some of the signs overlapping between models), it was presumed the sepsis scoring diagnostic tests were conditionally dependent when included in the same Bayesian analysis. The used WINBUGS codes were kindly provided by Prof. Dr. S. Buczinski (University of Montreal). The unknown parameters in the models were the sensitivity and specificity, the covariance in disease positive-animals (covDp), and covariance in disease-negative animals (covDn) of the sepsis scoring models. We included these covariances to model the dependencies between the tests. When establishing a BLCM model, prior information on the prevalence or the sensitivity or specificity of a diagnostic test can be included, using beta distributions (0 to 1), which we determined with Epitools (Ausvet) [24]. In this study, primarily, a model without any priors was conducted, followed by a model with prevalence of bacteremia (30%; 5th/95th percentile 0.8) in sick calves [1,3,6], and lastly a model including priors on prevalence of sepsis and on the sensitivity (80%; 5th/95th percentile 0.95) of blood culture testing [22,25,26]. These priors were supported by evidence from multiple studies and were therefore likely to improve the reliability and robustness of the model’s performance. The specific beta distributions of these priors are detailed in Tables 1–4. After a burn-in of 5000, 100,000 iterations were run for each model, while thinning was set at 1. The Dendukuri and Joseph parametrization was employed to expedite model convergence by restricting the prior to positive covariance between tests. Model convergence was assessed using density and Gelman–Rubin plots, while chain autocorrelation plots were reviewed to determine the necessity of chain thinning. For each parameter, the median and 95% credibility intervals (95% CI) were obtained. A sensitivity analysis for robustness was conducted by running alternative models with various extreme prior specifications. The median estimates were verified to fall within the 95% credibility interval (95% CI). Model fit was compared using the deviance information criterion (DIC).

Next to their sensitivity and specificity, performance of the test was reflected with their positive and negative predictive value (PPV and NPV, calculated using Winepi), the positive and negative likelihood ratio (LR+ and LR−), and the Youden Index (YI) [27].

## 3. Results

### 3.1. Study Sample

The final database compromised 131 sick calves. Among them, 71 (54.2%) were Belgian blue, 43 (32.8%) were Holstein Friesian, 13 (9.9%) were Belgian Blue and Holstein Friesian crossbreeds, 3 (2.3%) were Blonde d’Aquitaine, and 1 (0.8%) was a Jersey calf. The database included 67 male calves, 62 female calves, and 2 calves of unknown sex.

Mean and median age were 10.6 ± 10.0 and 9 days (min–max, 0–71). Mean and median temperature were 38.8 ± 1.27 and 38.9 °C (min–max, 32–41.7). Mean and median heart frequency were 129.8 ± 32.9 and 128 beats per minute (min–max, 48–204), and mean and median respiratory rate were 56.9 ± 32.5 and 48 breaths per minute (min–max, 8–168).

Regarding clinical conditions, 97 calves (74.0%) presented with diarrhea, while 34 (26.0%) did not. Pneumonia was detected on ultrasound in 50 calves (38.2%), while 24 calves (18.3%) displayed imaging indicative of neonatal respiratory distress syndrome on ultrasound (diffuse comet tails). Omphalitis was identified in 45 calves (34.3%) through palpation and/or ultrasound, with 29 cases classified as mild. Comorbidities, including pneumonia, omphalitis, and/or enteritis, were observed in 42 calves (32.1%).

In terms of prior antimicrobial treatment, 69 calves (52.7%) received no prior antimicrobial treatment from either an ambulatory veterinarian or farmer, while 57 (43.5%) had. Information on prior therapy was unavailable in five calves.

Of the calves, 101 (77.1%) fulfilled two or more SIRS-criteria. Abnormal state was present in 83 (63.4%) of the calves. Sepsis was diagnosed in 59 (45.0%), 35 (26.7%), 73 (55.7%), 62 (47.3%), and 72 (55.0%) calves according to the Trefz score, the Fecteau score, CSS, CSS_A_, and CSS_B_, respectively. A relevant positive blood culture was found in 29 calves (22.1%), while 9 positive blood cultures were classified as a contaminant. Bacteria isolated from the relevant blood cultures were: *Escherichia coli* (14), *Trueperella pyogenes* (3), *Mannheimia haemolytica* (2), *Salmonella enterica* (2), *Streptococcus uberis* (3), *Streptococcus ruminantium* (2), *Klebsiella oxytoca* (1), *Bibersteinia threhalosi*, and, in one, the combination of *S. enterica* + *E. coli* (1).

### 3.2. Bayesian Latent Class Models

As the sepsis scores all contain clinical information, the sepsis scoring models were considered dependent, and for this reason only results of dependent BLCM models are shown in this manuscript. Table 1 displays the three dependent BLCM models evaluating blood culture, and the Trefz and Fecteau scores. In models 2 and 3, prior information was added. The authors agreed on dependent BLCM model 3 as the final model, as this contained valuable prior information from earlier research and the credibility intervals converged more for this model. The sensitivity analysis showed little influence when adding extreme prior information to model 3. The sensitivity of sepsis detection in this population was highest for the Trefz score, and was similar for the Fecteau score and bacteremia detection. Specificity appeared highest for blood culture followed by the Fecteau score and the Trefz score. The PPV and NPV in this Bayesian analysis were, respectively, 50% and 76% for bacteremia, the LR+ and LR− were 2.4 and 0.73, and the YI was 0.23. The PPV and NPV of the Trefz score were, respectively, 44% and 83%, the LR+ and LR− were 1.9 and 0.48, and the YI was 0.33. The PPV and NPV of the Fecteau score were, respectively, 43% and 75%, the LR+ and LR− were 1.7 and 0.79, and the YI was 0.17.

Table 2 displays the BLCM model evaluating bacteremia detection as well as the Fecteau score and CSS among each other. The models with no priors and priors on only sepsis prevalence were made. For similar reasons as outlined for the Bayesian evaluation in Table 1, the model incorporating priors on sepsis prevalence and blood culture sensitivity was selected as the final model. The sensitivity of sepsis detection in this population was highest for CSS, followed by blood culture detection and the Fecteau score. Specificity was highest for bacteremia, followed by the Fecteau score and CSS. The predictive values and likelihood ratios are shown in Table 2. The YI was 0.39, 0.12, and 0.47 for blood culture, the Fecteau score, and the CSS, respectively.

Table 3 and Table 4 display the BLCM results evaluating bacteremia detection with blood culture, versus the Trefz score, versus CSS_A_ (Table 3), and versus CSS_B_ (Table 4). CSS_A_ and CSS_B_ were proposed as two alternative adapted models for CSS in practice. Both CSS_A_ and CSS_B_ were substantially more sensitive in comparison with the Fecteau score model. Bacteremia detection with blood culture was more specific compared to CSS_A_. Both blood culture and the Fecteau score were more specific compared to CSS_B_. The YI was 0.47 for CSS_A_ and 0.44 for CSS_B_.

The Fecteau score classified 20 out of 29 blood culture-positive calves as negative, while the Trefz score classified 13 out of 29 as negative. For CSS, CSSA, and CSSB, 6, 9, and 7 out of 29 blood culture-positive calves were classified as negative, respectively.

## 4. Discussion

The objective in this study was to evaluate existing and novel sepsis scoring systems and blood culture as diagnostic tests for sepsis in sick calves. This was conducted using Bayesian latent class modelling.

As stated in previous research, we are in need of a screening method for sepsis in calves with a high sensitivity to avoid missing septic calves and not treating them [5]. Afterwards, a confirming diagnostic test (e.g., biomarker) with a high specificity can be performed to not overuse (critically important) antimicrobials. The main finding in this study was that the models including the combination of ≥2 SIRS criteria and abnormal mental state had a higher sensitivity to diagnose sepsis in comparison to other sepsis scoring models. When only ≥2 SIRS criteria are used as diagnostic tool for sepsis without extra additional clinical parameters, sensitivity is higher, but too many false positive calves would be included. For instance, healthy excited calves can have both a high respiratory rate and heart frequency. Abnormal mental state was therefore included, as this is incorporated in screenings for sepsis in human medicine and abnormal behavior was demonstrated to be linked with bacteremia in calves as well [5,7,18]. When looking into the performance of the different suggested sepsis scores, the first Bayesian analysis comparing the Fecteau score, the Trefz score, and blood culture (Table 1) showed an overlap in the credibility intervals of the three diagnostic tests, thus no definite conclusions on significant differences between the three tests could be made. However, when an abnormal mental state was included (model CSS, CSS_A_, and CSS_B_) in the second, third, and fourth Bayesian analysis, a substantial difference between the sepsis scoring systems was present. Although there was still overlap in CI of some sensitivities and specificities (mainly due to a broad CI of bacteremia), the Fecteau score appeared less sensitive compared to the other sepsis scoring models. According to the authors, there are multiple possible explanations for this. The first one is that the models including SIRS and behavior perform a broader initial screening, looking less into specific details (such as exact fecal scoring and umbilical scoring) compared to the Fecteau score. Another explanation is the difference in the population on which the Fecteau score was constructed, i.e., in the study by Fecteau et al. [1], it was built on a population of solely diarrhetic calves. Nonetheless, in our study population, only 26% of the calves had no diarrhea, so this difference in population likely does not have a huge impact. A third explanation can be that the Fecteau score model was created for practitioners in the field, and presumably the clinical condition of most of the animals in our study population was relatively worse (clinic setting, a lot of critically ill animals) compared to that study. The authors do emphasize that not only calves with diarrhea are at risk for sepsis, as demonstrated in earlier research; thus, expanding the population to all sick calves might be most realistic for sepsis patients [5,6].

The models proposed in this manuscript could be interesting for their use as sepsis screening tests. According to the authors, the model **CSS** (combination of SIRS-criteria and abnormal mental state) is the most interesting in a hospital setting as it has a sensitivity of 86%. However, model **CSS**_B_, which is similar to **CSS** but substitutes abnormal leukocyte count for abnormal mucosal color, is an acceptable alternative for practice as its sensitivity remains 84%. In addition, another important reason to choose this model is that the white blood cell count is heavily affected by age in calves [28]. This indicates that using suggested cut-offs for all calves might not be ideal, but working with multiple cut-offs would be impractical. The advantage of using the CSS_B_ screening model is its immediate applicability, enabling rapid sepsis diagnosis while rationalizing the use of critically important antimicrobials. Additionally, in this economically driven sector, the model provides a cost-effective alternative to expensive diagnostic methods, such as blood cultures or extensive blood examinations. Although blood culture appeared to have the highest specificity (83–91%), it is not recommended as a diagnostic test for confirmation of sepsis in calves due to practical limitations and time constraints, unless rapid blood culture testing is available. It is worth emphasizing that only culture results of assumed true pathogens were classified positive as bacteremia, in accordance with the guidelines outlined in Section 2.3.1, thus requiring sterile double blood culture sampling [22]. This method is labor intensive and costly, thus the authors conclude that bacteremia detection is not suitable as a fast confirmation test for sepsis in calves due to both time constraints and practical limitations. However, blood culture remains crucial for etiological diagnosis and antimicrobial susceptibility results.

The specificity of the Fecteau score (77–78%) is fair but might not be high enough to serve as a confirmation test. The authors suggest to opt for other confirmation tests to reduce false positives, such as hypoglycemia < 59 mg/dL or hyperlactatemia > 7.7 mmol/L as suggested in previous research [5]. However, future research may potentially identify more accurate confirmation tests. That the investigated sepsis models do not serve as a proper confirmation tool, but rather as a screening tool, is further demonstrated by high NPVs and low PPVs. This implies that while calves with sepsis are less likely to go undetected, there is a bigger risk of overdiagnosis. According to the authors, the misclassification cost would be greater if it was the other way around (low NPV and high PPV), as calves requiring sepsis treatment would be missed, likely resulting in their death. From the animal welfare perspective, the latter seems worse than giving antimicrobials to a calf that does not have sepsis but likely has another condition requiring antibiotics. Although PPV and NPV provide information about post-test probability, their variability due to disease prevalence makes them less reliable than LR. For instance, in a population with a higher sepsis prevalence, the PPV would increase. The LR results further confirm that the tested models are unsuitable as confirmation tests for sepsis. This is demonstrated by the fact that none of the models had a LR+ > 10 or LR− < 0.1, the threshold values used to indicate a large change in post-test probability of disease. Only blood culture testing had a LR+ between 5–10 in the second Bayesian analysis (LR+ was 5.5, shown in Table 2), which reflects a moderate increase in post-test probability [29].

A limitation of this study is that despite the fact that sufficient (>103) samples were incorporated in the analysis, according to Bujang and Adnan et al. [20], there seems too little power to narrow the credibility intervals, making significant differences between diagnostic tests impossible in Table 1. For blood culture, the minimum sample size positive for disease (N1) was narrowly missed (29 instead of 31), but for all other tests, more than sufficient positive samples were present [20]. In the models in Table 2, Table 3 and Table 4, substantial differences could be demonstrated, indicating that when the disparity between tests was large enough, it could be detected statistically. A second limitation is that a large part of the study population was Belgian Blue calves, making the extrapolation to other study population more precarious. A third important limitation is that nearly half of the included calves received prior antimicrobial therapy before blood culture sampling. Although previous research demonstrated that (after excluding contaminants), prior antimicrobial use does not significantly affect the detection of bacteria in bacteremic calves, it has to be taken into account that the prior treatment can influence our analysis [22]. Therefore, an important final limitation was the lack of validation of the new scores on a second dataset, as assembling a database of (critically) ill calves in which all the required diagnostic tests were performed proved to be challenging. Thus, we suggest that currently described screening models should be evaluated on new datasets in the future to better estimate their value.

## 5. Conclusions

In this study population, the existing sepsis scores demonstrated insufficient sensitivity to function as a screening test for identifying calves with sepsis, being high-priority candidates for broad-spectrum antimicrobial therapy. Our novel proposed calf sepsis screening (**CSS**) model, assessing the combination of SIRS-criteria together with an abnormal mental state, seems promising as a screening tool for sepsis in calves. An alternative sepsis model (**CSS**_B_), in which abnormal leukocyte count was substituted for abnormal mucosae, holds potential for calf-side use in practice. For proper use, these screening tools need to be followed by a more specific diagnostic test to confirm sepsis. Validation of these tools on other populations of calves still needs to be conducted.

## Figures and Tables

**Table 1:** BLCM including information on posterior median and 95% credible interval values for conditional dependent BLCMs for the sensitivity and specificity for diagnosing sepsis with two different existing sepsis scoring models (the Trefz score and the Fecteau score) versus blood culture positivity (with a relevant bacterium).

Test		Model 1: No Priors ^1^		Model 2: Priors on Sepsis Prevalence ^2^		Model 3: Priors on Sepsis Prevalence and Sensitivity Blood Culture ^3^	
		*Prior Density*	*Posterior Density* *(Median % [95% CI])*	*Prior Density*	*Posterior Density* *(Median % [95% CI])*	*Prior Density*	*Posterior Density* *(Median % [95% CI])*
**Blood culture**	Se	Beta (1, 1)	28.3 (5.7–86.3)	Beta (1, 1)	30.5 (5.4–84.7)	Beta (2.366, 1.3406)	39.7 (15.0–90.3)
	Sp	Beta (1, 1)	79.9 (25.6–96.0)	Beta (1, 1)	80.9 (53.3–95.7)	Beta (1, 1)	82.8 (65.4–96.5)
**Trefz score**	Se	Beta (1, 1)	57.9 (11.9–97.1)	Beta (1, 1)	63.7 (11.4–97.7)	Beta (1, 1)	69.6 (18.1–98.1)
	Sp	Beta (1, 1)	59.7 (9.2–95.3)	Beta (1, 1)	61.5 (18.0–94.9)	Beta (1, 1)	62.6 (29.5–93.9)
**Fecteau score**	Se	Beta (1, 1)	33.8 (6.5–90.8)	Beta (1, 1)	37.0 (6.0–90.7)	Beta (1, 1)	39.3 (7.4–91.2)
	Sp	Beta (1, 1)	75.9 (23.8–96.5)	Beta (1, 1)	77.1 (47.1–96.8)	Beta (1, 1)	77.2 (55.1–96.0)
**Prevalence**		Beta (1, 1)	36.7 (2.2–96.0)	Beta (1.5385, 2.2565)	32.4 (4.3–81.1)	Beta (1.5385, 2.2565)	26.8 (3.5–75.3)
**covDp**		U (0, a)	0.005 (−0.21–0.12)	U (0, a)	−0.001 (−0.21–0.13)	U (0, a)	−0.004 (−0.19–0.12)
**covDn**		U (0, b)	0.02 (−0.20–0.12)	U (0, b)	0.02 (−0.09–0.12)	U (0, b)	0.03 (−0.05–0.12)
**DIC**			32.8		37.1		38.3

**Abbreviations**: CI, credibility interval; Se, sensitivity; Sp, specificity; covDn, covariance for negatives; covDp, covariance for positives; DIC, deviance information criterion. **Trefz score**: clinical scoring model for sepsis based on Trefz et al. [19], **Fecteau score**: clinical scoring model for sepsis based on Fecteau et al. [1]. ^1^ Model 1: no informative priors. ^2^ Model 2: informative priors on prevalence of sepsis in calves [1,3,6]. ^3^ Model 3: informative priors on prevalence of sepsis in calves and on sensitivity of blood culture [22,25,26].

**Table 2:** BLCM including information on posterior median and 95% credible interval values for conditional dependent BLCMs for the sensitivity and specificity for diagnosing sepsis with one existing sepsis clinical scoring model (the Fecteau score), a new constructed score systemic (calf sepsis screening, CSS), and blood culture positivity (with a relevant bacterium).

Test		Model with Priors on Sepsis Prevalence and Sensitivity Blood Culture ^3^					
		*Prior Density*	*Posterior Density* *(Median % [95% CI])*	*Predictive Value (%)*		*Likelihood* *Ratio*	
**Blood culture**	Se	Beta (2.366, 1.3406)	47.9 (18.9–92.6)	PPV	70	LR+	5.5
	Sp	Beta (1, 1)	91.3 (70.0–99.2)	NPV	80	LR−	0.57
**Fecteau score**	Se	Beta (1, 1)	34.5 (11.2–60.0)	PPV	39	LR+	1.5
	Sp	Beta (1, 1)	77.2 (61.8–96.0)	NPV	73	LR−	0.84
**CSS**	Se	Beta (1, 1)	86.4 (47.2–98.9)	PPV	49	LR+	2.2
	Sp	Beta (1, 1)	61.0 (31.7–93.7)	NPV	91	LR−	0.22
**Prevalence**		Beta (1.5385, 2.2565)	36.1 (10.8–67.8)				
**covDp**		U (0, a)	−0.015 (−0.11–0.073)				
**covDn**		U (0, b)	0.067 (−0.018–0.15)				
**DIC**			38.7				

**Abbreviations**: CI, credibility interval; Se, sensitivity; Sp, specificity; covDn, covariance for negatives; covDp, covariance for positives; DIC, deviance information criterion; PPV, positive predictive value; NPV, negative predictive value; LR+, positive likelihood ratio; LR−, negative likelihood ratio. **Fecteau score**: clinical scoring model for sepsis based on Fecteau et al. [1], **CSS**: new clinical scoring model including SIRS-criteria and an abnormal mental state. ^3^ Model with informative priors on prevalence of sepsis in calves [1,3,6] and on sensitivity of blood culture [22,25,26].

**Table 3:** BLCM including information on posterior median and 95% credible interval values for conditional dependent BLCMs for the sensitivity and specificity for diagnosing sepsis with one existing sepsis clinical scoring model (the Fecteau score), an adapted version of the new constructed clinical score (calf sepsis screening A, CSS_A_) for use in practice, and blood culture positivity (with a relevant bacterium).

Test		Model with Priors on Sepsis Prevalence and Sensitivity Blood Culture ^3^					
		*Prior Density*	*Posterior Density* *(Median % [95% CI])*	*Predictive Value (%)*		*Likelihood* *Ratio*	
**Blood culture**	Se	Beta (2.366, 1.3406)	46.0 (18.7–91.9)	PPV	63	LR+	4.0
	Sp	Beta (1, 1)	88.4 (69.5–98.6)	NPV	79	LR−	0.61
**Fecteau score**	Se	Beta (1, 1)	35.0 (9.7–69.4)	PPV	39	LR+	1.5
	Sp	Beta (1, 1)	76.8 (61.1–94.6)	NPV	73	LR−	0.85
**CSS_A_**	Se	Beta (1, 1)	79.9 (33.4–98.6)	PPV	51	LR+	2.4
	Sp	Beta (1, 1)	67.3 (37.0–96.5)	NPV	89	LR−	0.30
**Prevalence**		Beta (1.5385, 2.2565)	32.9 (7.8–67.6)				
**covDp**		U (0, a)	−0.020 (−0.14–0.074)				
**covDn**		U (0, b)	0.038 (−0.032–0.12)				
**DIC**			39.7				

**Abbreviations**: CI, credibility interval; Se, sensitivity; Sp, specificity; covDn, covariance for negatives; covDp, covariance for positives; DIC, deviance information criterion; PPV, positive predictive value; NPV, negative predictive value; LR+, positive likelihood ratio; LR−, negative likelihood ratio. **Fecteau score**: clinical scoring model for sepsis based on Fecteau et al. [1], **CSS_A_**: new clinical scoring model for practitioners including reduced SIRS-criteria (temperature, respiratory rate, heart frequency) and an abnormal mental state. ^3^ Model with informative priors on prevalence of sepsis in calves [1,3,6] and on sensitivity of blood culture [22,25,26].

**Table 4:** BLCM including information on posterior median and 95% credible interval values for conditional dependent BLCMs for the sensitivity and specificity for diagnosing sepsis with one existing sepsis clinical scoring model (the Fecteau score), an adapted version of the new constructed clinical score (calf sepsis screening B, CSS_B_) for use in practice, and blood culture positivity (with a relevant bacterium).

Test		Model with Priors on Sepsis Prevalence and Sensitivity Blood Culture ^3^					
		*Prior Density*	*Posterior Density* *(Median % [95% CI])*	*Predictive Value (%)*		*Likelihood* *Ratio*	
**Blood culture**	Se	Beta (2.366, 1.3406)	44.4 (17.0–91.6)	PPV	63	LR+	3.9
	Sp	Beta (1, 1)	88.7 (67.7–98.7)	NPV	79	LR−	0.63
**Fecteau score**	Se	Beta (1, 1)	35.5 (10.2–71.2)	PPV	40	LR+	1.6
	Sp	Beta (1, 1)	77.5 (60.5–95.6)	NPV	74	LR−	0.85
**CSS_B_**	Se	Beta (1, 1)	84.2 (38.2–99.0)	PPV	47	LR+	2.1
	Sp	Beta (1, 1)	59.7 (26.3–94.3)	NPV	90	LR−	0.26
**Prevalence**		Beta (1.5385, 2.2565)	35.0 (7.6–70.6)				
**covDp**		U (0, a)	−0.001 (−0.12–0.084)				
**covDn**		U (0, b)	0.044 (−0.033–0.13)				
**DIC**			38.8				

**Abbreviations**: CI, credibility interval; Se, sensitivity; Sp, specificity; covDn, covariance for negatives; covDp, covariance for positives; PPV, positive predictive value; NPV, negative predictive value; LR+, positive likelihood ratio; LR-, negative likelihood ratio. **Fecteau score**: clinical scoring model for sepsis based on Fecteau et al. (1997), **CSS_B_**: new clinical scoring model for practitioners including adapted SIRS-criteria (temperature, respiratory rate, heart frequency, mucosal color) and an abnormal mental state. ^3^ Model with informative priors on prevalence of sepsis in calves [1,3,6] and on sensitivity of blood culture [22,25,26].

## Data Availability

The original contributions presented in the study are included in the article/Supplementary Materials, further inquiries can be directed to the corresponding author.

## Supplementary Materials

The following supporting information can be downloaded at: https://www.mdpi.com/article/10.3390/ani15040586/s1, Figure S1. Visual representation of the adapted calf screening score (CSS_A_) for application in first-line practice. SIRS-criteria were adapted. Abnormal white blood cell count was excluded from the SIRS-criteria, as it is no calf-side test. The combination of ≥2 adapted SIRS criteria and abnormal mental state are considered sepsis-suspected calves according to this screening score.
