# Supplementary material for: Optimization of Antimicrobial Use for Sepsis in Calves: Bayesian Evaluation of Existing and Novel Sepsis Scores
Source: Animals (Basel). 2025 Feb 18;15(4):586. doi: 10.3390/ani15040586 (PMC11851423; doi:10.3390/ani15040586)
Supplement: Supplementary file 1 [file animals-15-00586-s001.zip › animals-3398323-supplementary.pdf]

animals MDPI

Supplementary information

Optimization of Antimicrobial Use for Sepsis in Calves: Bayesian Evaluation of Existing and Novel Sepsis Scores

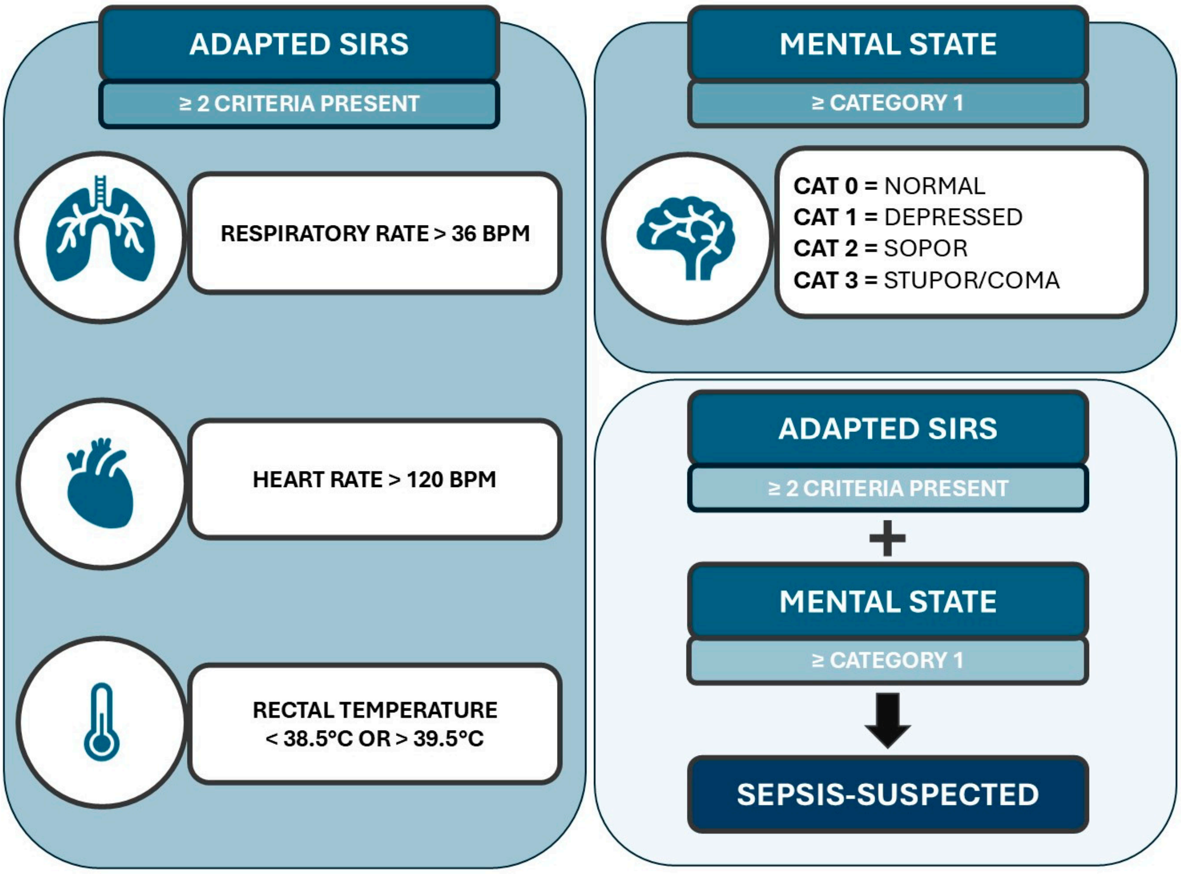

Figure S1. Visual representation of the adapted calf screening score (CSS_A) for application in first-line practice. SIRS-criteria were adapted. Abnormal white blood cell count was excluded from the SIRS-criteria, as it is no calf-side test. The combination of ≥2 adapted SIRS criteria and abnormal mental state are considered sepsis-suspected calves according to this screening score.
